# Mental Recreation

**Published:** 1871-12

**Authors:** 


### MENTAL RECREATION.

MENTAL diversion, mental exhilaration, mental release from the cares and business and worries of life, are not only essential to healthful thought, to a healthy condition of the mind, but they materially promote bodily vigor and physical well being. Amusements should more largely enter into American family life than they now do; it is the absence of it to too great an extent, which leads to many social evils, to many habits and practices which ruin the health and the morals of our sons and daughters in a great many cases. If boys and girls from fifteen and upwards do not find amusements at home, especially during the long winter evenings, they sigh for places where exhilaration can be found: the sons for the street — the daughters for the dance, for the theatre, for visiting those of their associates whose homes are more lively.

Young people cannot be expected to have books and newspapers always in their hands, or sit demurely by the family fireside by the hour, in hemming and stitching and knitting. Games and pastimes should be more freely introduced into our families; there should be more of off-hand visiting; of informal calls; where one neighbor can drop into another neighbor's house after dark, and spend an hour or two in unrestrained social intercourse, without form or ceremony, for two or three nights in every week; thus giving air, exercise, and recreation.

### A NEW DIVERTISEMENT

was inaugurated last winter, which promises to be repeated, and ought to find very general favor among the best classes of society; it is much to be regretted that it was not sooner thought of and practiced upon. In December, 1870, that prince of lecturers, Dr. John Lord, LL. D., began a course of Biographical and Historical lectures, twenty-five in number, on the characters of some of the most eminent men and women of a bygone age, — Bacon, Bonaparte,

Luther, Madame De Staël, and others ; they were delivered at midday, two or three a week, extending through several months, in Association Hall, New York city; and it can be said without hesitation, that a more delightfully instructive course of lectures has never been delivered in any previous winter. All the surroundings were most agreeable. The large, well lighted and well heated hall of the Young Men's Christian Association Building was admirably adapted for the purpose; the character of the audience was itself inspiring, as it comprised the fashion, the culture, and the intellect of the city, — men, women, and maidens. Rain or shine, sleet, hail, or storm, all the same, the building was crowded by admiring and intensely interested hearers; and we will venture to say, that the very memory of these lectures of Dr. Lord, last winter, is fraught with pleasure and with love; and we look back upon the time with associations mournfully pleasing.

Then with it all was the happy manner of the lecturer. He had grown gray since we last heard him, ever so many years ago! but there was the same genial face, the same merry twinkle of the eye, the same friendly smile, and the same quick step to the platform, and waking up voice and manner, in delightful contrast with that slow, sleepy, stately mien with which most men mount the rostrum. For ourself and the members of the family, we found that looking forward to a lecture day was a relief and a delight; to be prepared for beforehand, and talked about afterwards, to one another and to friends. Being delivered in the daytime, ladies could attend alone, when their husbands and brothers could not accompany them; and others, whose health would not allow of exposure to the winter night air, were able to take a noon-day walk; the preparation for which, and the scenes and sights on the way, altogether made it a form of exercise more invigorating and healthful than any medicine of the apothecary.

A most satisfactory feature is Dr. Lord's delivery; it is of itself a delight; every sentence, word, and syllable is enunciated with perfect distinctness; the voice is clear and sharp; the words leap out, they don't drawl; the enunciation does not go down into the cellar at the end of every sentence, and there is a tone and a life in the expression of every idea, which not only proves the speaker's sincerity, but his interest in the subject seems as lively as your own, and going to sleep, or even a nod, is impossible to any being of brains.

The Doctor partially promised to deliver another course if nothing happened, on an entirely new set of subjects, some twenty-five or thirty in number, during the present winter. There is no sort of use to urge the people to come, but we want to hint to the readers of the JOURNAL, that in order to get good seats on the lower floor, they would do well to secure their tickets just as soon as Mr. Putnam announces, at his elegant bookstore windows, corner of Twenty-third Street and Fourth Avenue, that the literary lion of the day has come with another intellectual treat, two or three times a week during the winter, to the culture and refinement of New York; and why may not, on the off days of the week, same place and hour, another course of lectures be inaugurated; a different man on different subjects every day, civil and literary, moral and religious; and to give a little spice to the whole, and make them take, bring some ladies to the front? We will take tickets to the course for ourself and for several at our elbow, to begin with.

